# Biomimetic Boron Nitride Nanoparticles for Targeted Drug Delivery and Enhanced Antitumor Activity

**DOI:** 10.3390/pharmaceutics15041269

**Published:** 2023-04-18

**Authors:** Hui Li, Wei Qiao, Yizhe Shen, Huashan Xu, Yuan Fan, Yuxiang Liu, Yadi Lan, Yan Gong, Fuxue Chen, Shini Feng

**Affiliations:** 1 School of Environmental and Chemical Engineering, Shanghai University, 333 Nanchen Road, Shanghai 200444, China; 2 School of Life Sciences, Shanghai University, 333 Nanchen Road, Shanghai 200444, China

**Keywords:** boron nitride, biomimetic, cancer cell membrane, homologous targeting, drug delivery

## Abstract

Boron nitride nanomaterials are being increasingly recognized as vehicles for cancer drug delivery that increase drug loading and control drug release because of their excellent physicochemical properties and biocompatibility. However, these nanoparticles are often cleared rapidly by the immune system and have poor tumor targeting effects. As a result, biomimetic nanotechnology has emerged to address these challenges in recent times. Cell-derived biomimetic carriers have the characteristics of good biocompatibility, long circulation time, and strong targeting ability. Here, we report a biomimetic nanoplatform (CM@BN/DOX) prepared by encapsulating boron nitride nanoparticles (BN) and doxorubicin (DOX) together using cancer cell membrane (CCM) for targeted drug delivery and tumor therapy. The CM@BN/DOX nanoparticles (NPs) were able to target cancer cells of the same type on its own initiative through homologous targeting of cancer cell membranes. This led to a remarkable increase in cellular uptake. In vitro simulation of an acidic tumor microenvironment could effectively promote drug release from CM@BN/DOX. Furthermore, the CM@BN/DOX complex exhibited an excellent inhibitory effect against homotypic cancer cells. These findings suggest that CM@BN/DOX are promising in targeted drug delivery and potentially personalized therapy against their homologous tumor.

## 1. Introduction

Nanoparticle-based drug delivery systems (DDS) have provided more choices for drug delivery in recent years [1,2,3]. Conventional nano-carriers include inorganic nanoparticles (NPs), polymers, micelles, dendrimers, liposomes, and more, which are designed to passively target to tumor sites by persistent enhanced permeability and retention (EPR) effects in the tumor environment [4,5,6,7]. However, the application of many DDS is still significantly limited due to premature drug leakage, limited tumor targeting, and instability in the circulation [2,8]. Biomimetic nanoparticles were designed to mimic the structure and function of natural biological entities, allowing them to interact with biological systems in a more natural and effective way. By utilizing natural materials, biomimetic nanoparticles can retain their biological features and functions [9,10]. Exosomes, as natural extracellular vesicles, play a crucial role in various biological processes, including immune responses, apoptosis, and intercellular communication [11,12]. As a result, the potential of exosomes as drug delivery vehicles was fully explored [11,13]. However, the development of exosome-based drug vectors remains challenging due to issues such as low production, high batch-to-batch variability, and drug loading difficulties [14]. Recently, researchers have successfully used cellular membrane material for NPs preparation, which not only avoids the limitations derived from extracellular vesicles but also offers the advantage of being able to completely replicate the surface antigenic diversity of source cells [15,16,17]. For instance, there are red blood cell membrane-coated NPs that are designed to circulate in the body for longer periods of time [18,19], mesenchymal stem cells membrane-camouflaged nanomaterials with cancer targeting abilities [20,21], and leukocyte membrane biomimetic NPs with the ability to traverse the endothelium [22,23]. In addition, cancer cell membranes have been selected as a coating for nanoparticles (NPs) due to their ability to recognize and attach to the same cell lines. This resulted in efficient internalization at the cellular level [24,25]. Cancer cells can navigate and anchor homologous cells by adhesion molecules on the membrane, promoting the accumulation of cell membrane vectors at homologous tumor sites [26,27,28]. The surface decoration with cancer cell membranes is considered promising in the application of NPs in vivo.

Among NPs, boron nitride (BN) nanomaterials have received widespread attention as a result of their excellent physical and chemical properties [29,30,31]. Due to its great biocompatibility, BN also showed a broad application prospect in drug delivery and cancer therapy [32,33]. In addition, BN was applied to boron neutron capture therapy (BNCT) for tumor treatment on account of its rich ^10^B content [34,35,36,37]. However, the surface hydrophobicity of boron nitride nanomaterial makes it easy to agglomerate in solvent, which leads to the reduction in the specific surface area and drug loading [38,39,40]. Our group previously used BN modified with folic acid as carriers for anticancer drug delivery, which significantly enhanced the cellular uptake and tumor inhibition efficacy [41]. It was also demonstrated that red cell membrane encapsulated BN exhibited superior mono-dispersity, an extended circulation period, and improved stability in a physiological environment [42]. Therefore, with reasonable surface modification, BN was a promising and potential candidate for efficient anticancer drug delivery.

**Figure 1:**
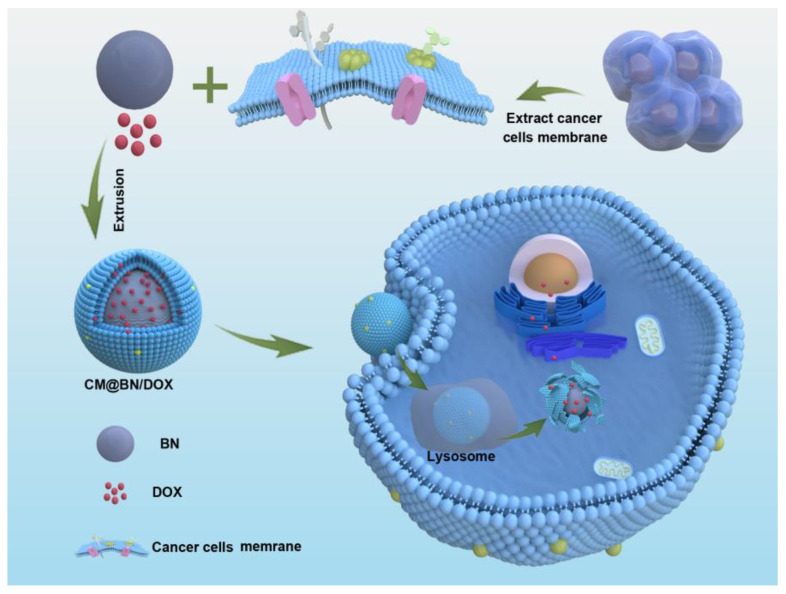
The preparation of CM@BN/DOX complexes.

In this study, we prepared cancer cell membrane-camouflaged bionic BN nanoparticles for targeted therapy of homologous tumors (Figure 1). Firstly, BN was mixed with doxorubicin (DOX) for shock. Then, BV2 cell membrane was collected and covered on the surface of BN by mechanical extrusion to form biomimetic nanoparticles with a core-shell structure. After centrifugation, CM@BN/DOX were obtained and characterized. Then, the research examined the DOX loading and pH-susceptibility drug release of CM@BN/DOX, while also verifying the cellular uptake and targeting ability of CM@BN/DOX to homotypic cells. Finally, the anticancer effects of CM@BN/DOX were investigated in detail.

## 2. Materials and Methods

### 2.1. Materials

Sangon Biotech (Shanghai, China) provided the following materials for our subject paper: a sodium dodecyl sulfate-polyacrylamide gel electrophoresis (SDS-PAGE) kit, doxorubicin (DOX), phenylmethylsulfonyl fluoride (PMSF), and 4′,6-diamidino-2-phenylindole (DAPI). Fetal bovine serum (FBS), trypsin-EDTA, Dulbecco’s modified Eagle’s medium (DMEM), and phosphate buffer saline solution (PBS) were bought from BI in Shanghai, China. Cell Counting Kit-8 (CCK-8), Calcein-AM/PI Double Staining Kit and Annexin V-Alexa Fluor 647/PI Apoptosis Detection Kit were outsourced from Do Jindo (Kumamoto, Japan). Coomassie brilliant blue was provided by Beyotime (Shanghai, China).

### 2.2. Cell Culture

The BV2 mouse glioma cells, GMI-R1 rat microglial cells, and HeLa human cervical carcinoma cells were outsourced from the Cell Bank of the Chinese Academy of Sciences in Shanghai, China. Astrocyte cells are extracted from the hippocampus of neonatal mice. Cells were incubated with DMEM high-sugar medium containing 10% FBS.

### 2.3. Preparation of Cancer Cell Membranes

The BV2 cells were incubated in cell culture dishes that were 10 cm in diameter. Then, the cells were collected and slightly rinsed three times with ice-cold PBS. The obtained cells were uniformly suspended in a hypotonic lysing buffer containing PMSF, which hindered the degradation of cell membrane proteins, and then placed at 4 °C for 24 h. The cells were disrupted by homogenizer for 20 passes. The homogenized solution of cell membrane fractions was centrifuged at 3200× *g* for 20 min. Cell membrane fragments were collected by ultracentrifuging the supernatant at 100,000× *g* for 30 min. A plasmalemma sediment was weighed 2 mg and resuspended in 1 mL of PBS buffer with pH of 7.4. The sample was stored at −80 °C for further experiments.

For the preparation of CM@BN, 2 mg of BN in 1 mL of PBS were dissolved. Then, CM and BN were mixed at a weight ratio of 1:1 and sonicated for 5 min by bath sonicator rated at 60 W. Subsequently, the mixture was added to a micro-extruder and polycarbonate films with pore sizes of 400 nm and 200 nm were extruded 20 times respectively. The resulting CM@BN was then subjected to centrifugation at 12,000 rpm for 20 min to remove excess membrane and BN.

### 2.4. Characterization

The morphology of BN and CM@BN were observed by transmission electron microscope (TEM, Thermo Scientific, Waltham, MA, USA). We utilized a K-Alpha+ X-ray photoelectron spectrometer from Thermo Scientific in order to conduct X-ray photoelectron spectroscopy (XPS) measurements. The hydrodynamic diameter and zeta-potential of CM, BN, CM@BN, and CM@BN/DOX were measured by dynamic light scattering (DLS) on a Zetasizer (Malvern, UK). The protein underwent analysis through sodium dodecyl sulfate-polyacrylamide gel electrophoresis (SDS-PAGE), while cells which exhibited fluorescence were observed by using confocal laser scanning microscopy (CLSM, Zeiss, Jena, Germany). Endocytosis and apoptosis were detected by Beckman’s flow cytometry (Beckman, Brea, CA, USA).

### 2.5. Biocompatibility Assay

BV2, HeLa, GMI-R1, and Ast the cells were cultivated at an initial concentration of 4000 cells/well in 96-well plates. After 24 h of incubation, the solution (10 µL) of DOX and CM@BN with a series of concentrations were added to each well. After co-incubating for 24 h, the culture medium was replaced with 100 µL of fresh medium. Next, 10 μL solution of CCK-8 was added, and the cells were placed in the incubator for 2 h. To determine the cell viability percentage, we utilized a microplate reader (Molecular Devices, San Jose, CA, USA) to obtain the optical density at 450 nm. The viability percentage of cells was performed using the following equation:

$$\mathrm{Cell}\;\mathrm{viability}\;(\%)=\frac{{\mathrm{OD}}_{450(\mathrm{sample})}}{{\mathrm{OD}}_{450(\mathrm{control})}}\times 100\%$$

where OD_450(sample)_ represents the absorbance of cells dealt with NPs, while OD_450(control)_ represents the absorbance of cells cultured with PBS.

To further assess the biocompatibility of free BN or CM@BN/DOX, we conducted an extracorporeal hemolysis assay by co-incubating them with red blood cells. To obtain red blood cells, fresh rat blood was centrifuged at 1500 rpm for 10 min and PBS was added to create a 2% erythrocyte suspension. Next, the resulting suspension (0.5 mL) was incubated with either free BN (0.5 mL) or CM@BN/DOX (0.5 mL) containing various BN concentrations (20, 40, 60, 80 and 100 µg/mL) at 37 °C for 4 h. The negative control group was created by co-incubating 0.5 mL of PBS with 0.5 mL of red blood cell suspension. The positive control group was created by mixing 0.5 mL of 0.1% Triton X-100 in PBS with 0.5 mL of red blood cell suspension. The suspension was then centrifuged at 3000 rpm for 5 min, and the optical absorbance at 570 nm of the supernatant was measured. To determine the hemolysis ratio, the formula below was utilized:

$$\mathrm{Hemolysis}\;\mathrm{ratio}\;(\%)=\frac{\mathrm{OD}\;(\mathrm{sample})-\mathrm{OD}\;(\mathrm{negative}\;\mathrm{control})}{\mathrm{OD}\;(\mathrm{positive}\;\mathrm{control})-\mathrm{OD}\;(\mathrm{negative}\;\mathrm{control})}\times 100\%$$

### 2.6. Drug Loading and Release

First, 1 mg of DOX in 1 mL of PBS was dissolved to prepare a 1 mg/mL DOX solution. Subsequently, CM and BN solution (2 mg/mL) were mixed together with the volume ratio of 1:1, then 1 mL of DOX solution was added. The mixture underwent sonication for 5 min before being extruded through polycarbonate porous membranes. The first 20 cycles were performed using membranes with pore sizes of 400 nm, followed by 20 cycles using membranes with pore sizes of 200 nm. To prepare BN/DOX, we mixed 1 mL of BN (1 mg/mL in PBS) with 1 mL of DOX solution (1 mg/mL). We then shook the mixture in the dark for 24 h at room temperature. Afterwards, the mixture was centrifuged at 12,000 rpm for 20 min and resuspended with PBS (pH 7.4). To determine the DOX loading capacity on BN, we calculated the concentration of DOX in the supernatant using a microplate reader. The drug loading capacity was determined using the formula:

$$\mathrm{Drug}\;\mathrm{loading}\;\mathrm{capacity}=\frac{\mathrm{Amount}\;\mathrm{of}\;\mathrm{DOX}\;\mathrm{loaded}\;\mathrm{on}\;\mathrm{carrier}}{\mathrm{Amount}\;\mathrm{of}\;\mathrm{carrier}}$$

During the DOX release experiments, we suspended CM@BN/DOX in 2 mL of PBS with varying pH levels of 7.4 or 5.0. The suspension was then shaken in the dark at room temperature. At specific time intervals, we collected 1 mL of supernatant and replaced it with an equivalent amount of fresh PBS. To measure the release of DOX, we used the microplate reader which set to 480 nm.

### 2.7. Cellular Uptake and Homologous Targeting

BV2 cells were planted in Petri dishes (4000 cells per dish) and incubated for 24 h. Free DOX, BN/DOX, or CM@BN/DOX were added to the dish and incubation continued for 2 h. The cells were first washed three times with PBS before being fixed with 4% paraformaldehyde. Following this, the nuclei were stained with a marker of nuclei named DAPI. Lastly, the fluorescence of DOX was observed through the use of CLSM.

BV2, HeLa, GMI-R1, and Ast cells were incubated for 24 h. The cells were incubated with CM@BN/DOX for 2 h. Subsequently, the cells were first washed three times with PBS before being fixed for 20 min. Then, the nuclei were labeled via DAPI and the fluorescence of DOX was observed.

For flow cytometry (FCM), the cells were dealt with free DOX, BN/DOX, or CM@BN/DOX for 2 h. Then, the cells were washed with PBS at a pH of 7.4. Afterwards, the fluorescence intensity produced by DOX was measured through using FCM.

### 2.8. Antitumor Activity

BV2, HeLa, GMI-R1, and Ast cells were cultivated for 24 h in 96-well plates (4000 cells per well). Then, CM@BN/DOX was added to 0–1 μg/mL of DOX series media. After 24 h of incubation, 10 μL of CCK-8 was added and incubated for another 2 h. The absorbance was measured at 450 nm.

$$\mathrm{Cell}\;\mathrm{viability}=\frac{A}{A_{0}}\times 100\%$$

where the OD_450_ of the treated cells is represented by A, while the OD_450_ of the untreated cells is represented by A_0_.

To perform the live/dead assays, BV2, HeLa, GMI-R1, and Ast cells were incubated in confocal dishes at a concentration of 1 × 10^4^ cells. After 24 h, CM@BN/DOX were added for another 24 h. Then, the cells were swilled three times and stained with calcein AM and PI. Eventually, the resulting fluorescence was then observed by use of CLSM.

The antitumor efficacy was further studied through flow cytometry. BV2, HeLa, GMI-R1, and Ast cells were seeded in 6-well plates and incubated for the entire night in a suitable environment. The cells were treatedwith CM@BN/DOX at equivalent concentration of DOX (1 μg/mL) for 24 h. After that, the cells were swilled and the apoptosis rates were determined by using the apoptosis kit and the results were analyzed through FCM.

### 2.9. Statistical Analysis

In this study, Student’s *t*-test was used for statistical analysis. The data were presented as mean ± standard deviation. Significance was determined with the following values: * *p* < 0.05, ** *p* < 0.01, and *** *p* < 0.005. Any differences that met these criteria were considered to be statistically significant.

## 3. Results and Discussion

### 3.1. Preparation and Characterization of CM@BN

**Figure 2:**
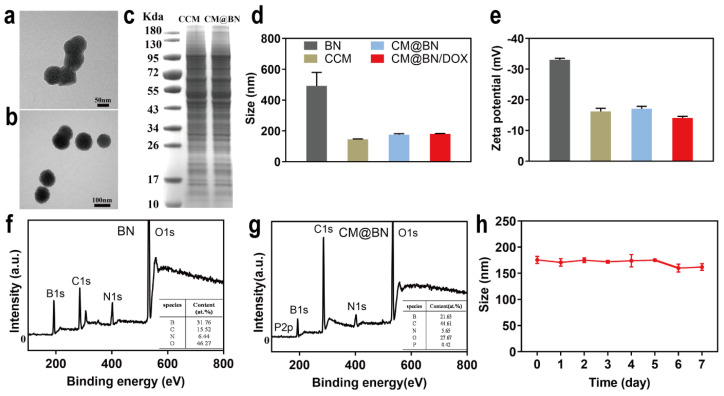
Characterization of CM@BN/DOX. (**a**) TEM image of boron nitride (BN). (**b**) TEM image of CM@BN. (**c**) The protein profiles of the BV2 cell membrane fragments and CM@BN. (**d**) Hydro-dynamic size of BN, CCM, CM@BN, and CM@BN/DOX. (**e**) Zeta potentials of BN, CCM, CM@BN, and CM@BN/DOX. (**f**) XPS spectrum of BN. (**g**) XPS spectrum of CM@BN. (**h**) Stability of CM@BN/DOX in PBS (pH = 7.4).

BN were synthesized through chemical vapor deposition as previously reported [43]. As shown in Figure 1, BV2 cell membranes were extracted and mixed with BN for physical extrusion, yielding cancer cell membrane-coated BN (CM@BN). The morphology and structure of CM@BN were characterized by TEM. Figure 2a demonstrated that BN possessed a consistent spherical shape with an average diameter of around 100 nm. After camouflage with cell membrane, CM@BN exhibited a core–shell structure with an average diameter of 175 nm as expected, and the outer BV2 cell membrane shell was approximately 10 nm in thickness (Figure 2b), which was obviously in agreement with the previous report [44,45]. It should be noted that after modification of cell membranes, the dispersibility of CM@BN solution was significantly improved, which may be conducive to ingestion of cancer cells. The surface protein of CM@BN from source cells was verified by SDS-PAGE (Figure 2c). Compared to natural BV2 cell membranes, CM@BN complexes were found to preserve a higher proportion of endogenous membrane proteins. Therefore, there is a reasonable prospect that the biological properties of CM will be transferred to CM@BN.

Compared to pristine BN, the hydrodynamic diameter of CM@BN and CM@BN/DOX was increased from 492 nm to 175 nm and 180 nm, respectively (Figure 2d). The significant decrease in size was due to the successful coating of the cell membrane, which was consistent with the results of the TEM. The zeta potential of CM@BN (−17 mV) was also compared to the original cancer cell membrane (−16 mV), but was much higher than the pristine BN (−33 mV) as shown in Figure 2e. Next, the chemical elements of nanoparticles were analyzed by XPS spectrum, the results of which revealed that P was present in CM@BN but absent in free BN (Figure 2f,g). These results suggested the successful translocation of natural cancer cell membrane onto the BN nanoparticles. Meanwhile, the long-term stability of CM@BN/DOX complexes in PBS (pH = 7.4) was detected and no obvious change in size was noted over a period of 7 d, indicating the suspension could be stored for an extended period without any noticeable aggregation or structural disruption at room temperature. (Figure 2h).

### 3.2. Biocompatibility Assay

**Figure 3:**
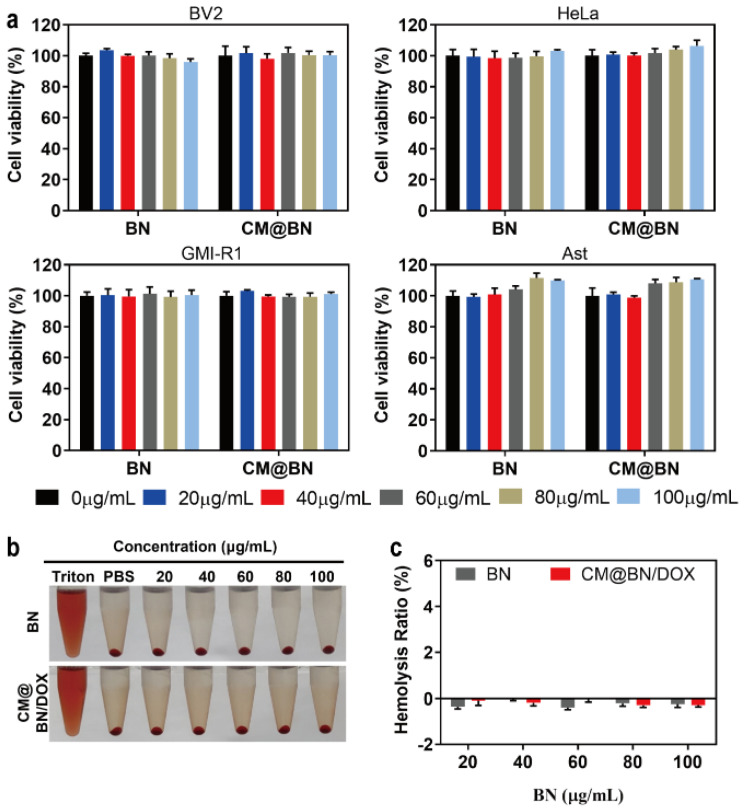
Biocompatibility in vitro. (**a**) The cytotoxicity of BN and CM@BN on various cell lines using concentrations ranging from 0 to 100 μg/mL for a 24-h period. (**b**,**c**) The hemolysis of red blood cells that were incubated with Triton X-100, PBS, BN, and CM@BN/DOX.

The basic biological properties of DDS, such as low toxicity, high biosafety, and excellent biocompatibility are prerequisites for their widespread and effective application [46,47]. Therefore, we evaluated the viability of cells (including BV2, HeLa, GMI-R1, and Ast cells) exposed to different concentrations of NPs by CCK-8. As indicated in Figure 3a, BN and CM@BN demonstrated low cytotoxicity to four different cells, even when the concentration reached 100 μg/mL. In addition, the hemolytic effect was evaluated by ex vivo incubation with erythrocytes. Both BN and CM@BN demonstrated no significant hemolysis rate (less than 5%) in concentrations of up to 100 μg/mL (Figure 3b,c). These results reflected the compatibility of CM@BN as a drug delivery carrier.

### 3.3. Drug Loading and Release

**Figure 4:**
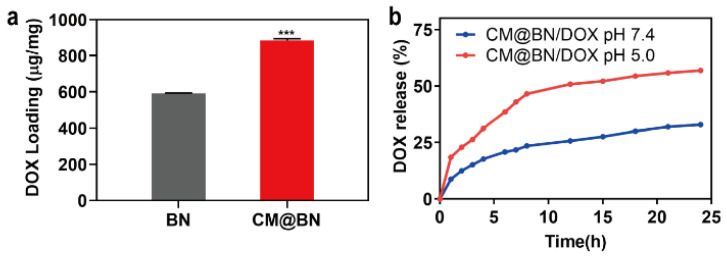
Drug loading and release. (**a**) Amount of doxorubicin (DOX) loaded on BN and CM@BN. (**b**) DOX release from BN/DOX and CM@BN/DOX complexes at different conditions. Data expressed as means ± SD, *** *p* < 0.001.

In an effort to achieve effective drug delivery in tumor treatment, it is crucial for carriers to stably encapsulate a large number of anticancer drugs. To validate the drug loading capacity of biomimetic NPs, DOX, a chemotherapeutic drug that has been routinely used to treat several types of tumors was set as a model drug to load onto BN and CM@BN. Figure 4a revealed that the loading amount of DOX on BN was 592.3 μg/mg, while it was 884.4 μg/mg on CM@BN. It was hypothesized that DOX was not only adsorbed onto the surface of BN, but also diffused through the membrane bilayer. Additionally, the mechanical extrusion was also found to enhance drug loading. Then, the drug release behavior of CM@BN/DOX was evaluated under different pH conditions. As shown in Figure 4b, CM@BN/DOX exhibited indiscernible drug release in a neutral environment (pH 7.4). However, in an acidic environment (pH 5.0), CM@BN/DOX displayed accelerated persistent release behavior during the period of 24 h. After 24 h, CM@BN/DOX released 60.2% of DOX at pH 5.0 compared to only 32.6% at pH 7.4. The release profile suggests that CM@BN/DOX have ability to remain stable under physiological conditions and effectively release drugs in acidic surroundings. This excellently resolves the premature release of drug, thereby reducing the side effects of therapeutics.

### 3.4. In Vitro Cellular Uptake and Targeting Ability

**Figure 5:**
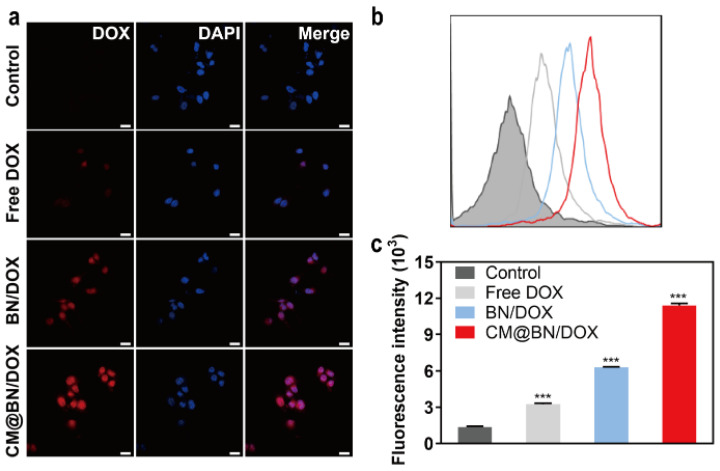
Cellular uptake. (**a**) Confocal laser scanning microscopy (CLSM) images of BV2 cells co-cultured with free DOX, BN@DOX, and CM@BN/DOX for 2 h. Scale bar = 20 µm. (**b**) Analysis of free DOX, BN/DOX, and CM@BN/DOX uptake by BV2 cells by flow cytometry (FCM). (**c**) Quantitative analysis of cellular uptake by FCM. Data are expressed as means ± SD, *** *p* < 0.001.

Doxorubicin acts on cancer cells by intercalating into cancer cells’ DNA, which disrupts topoisomerase II-mediated DNA repair. Additionally, it generates free radicals that cause damage to DNA, proteins, and cellular membranes [48,49,50]. Whether the drug can be efficiently ingested by tumor cells determines its efficiency. Thus, the cellular uptake of free DOX, BN/DOX, and CM@BN/DOX in BV2 cells were investigated. As indicated in Figure 5a, the fluorescence of cells treated with free DOX was the weakest. This might be due to the fact that the DOX molecule was transported into cells through passive diffusion and could be easily eliminated by P-gp [51]. The fluorescence intensity in the CM@BN/DOX group was higher than that of the free DOX and BN/DOX groups. It was supposed that BN/DOX and CM@BN/DOX was internalized into cells though the endocytosis. In addition, the membrane shell structure of CM@BN/DOX prevented drug leakage and efflux during transportation. The quantitative analysis of these results were verified by FCM (Figure 5b,c). These results demonstrated that the cell membrane coating facilitated the cellular internalization of biomimetic NPs by BV2 cells.

**Figure 6:**
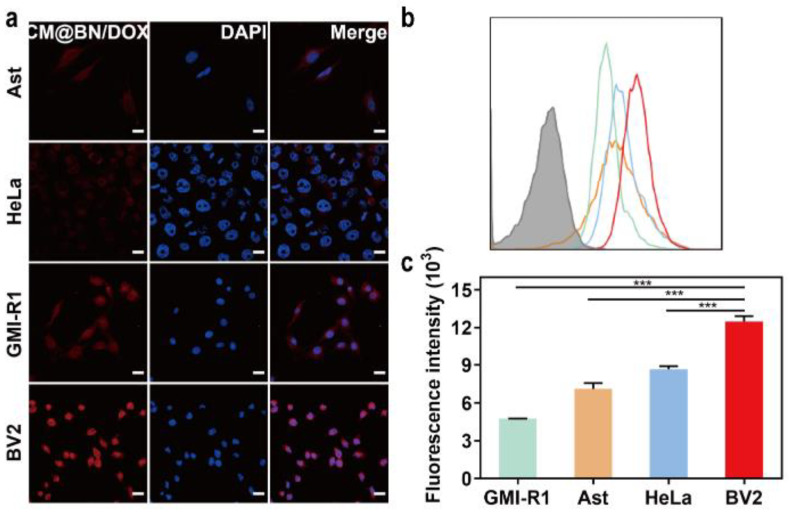
Homologous targeting of CM@BN/DOX. (**a**) Confocal laser scanning microscopy images of BV2, HeLa, GMI-R1, and Ast cells incubated with CM@BN/DOX for 2 h. Scale bar = 20 µm. (**b**) The intracellular uptake of CM@BN/DOX by BV2, HeLa, GMI-R1, and Ast cells using FCM (blank group was represented as grey). (**c**) Quantitative analysis. Data are presented as means ± SD, *** *p* < 0.001.

Subsequently, the homologous targeting ability of cancer cell membrane camouflaged NPs was compared in BV2, HeLa, GMI-R1, and Ast cells. The results of the cellular uptake of the CM@BN/DOX as shown in Figure 6a demonstrated that BV2 cells incubated with homologous cytomembrane encapsulated CM@BN/DOX displayed the highest fluorescence signal among all the experimental groups. Figure 6b and c confirmed similar results as those obtained from the previous experiment. The results from FCM indicated that BV2 cells that were treated with CM@BN/DOX displayed a more intense fluorescence compared to the other cells. These results demonstrated the fabricated biomimetic NPs remained the homologous targeting properties gained from cancer cell membrane and significantly promoted cellular endocytosis.

### 3.5. In Vitro Antitumor Activity

**Figure 7:**
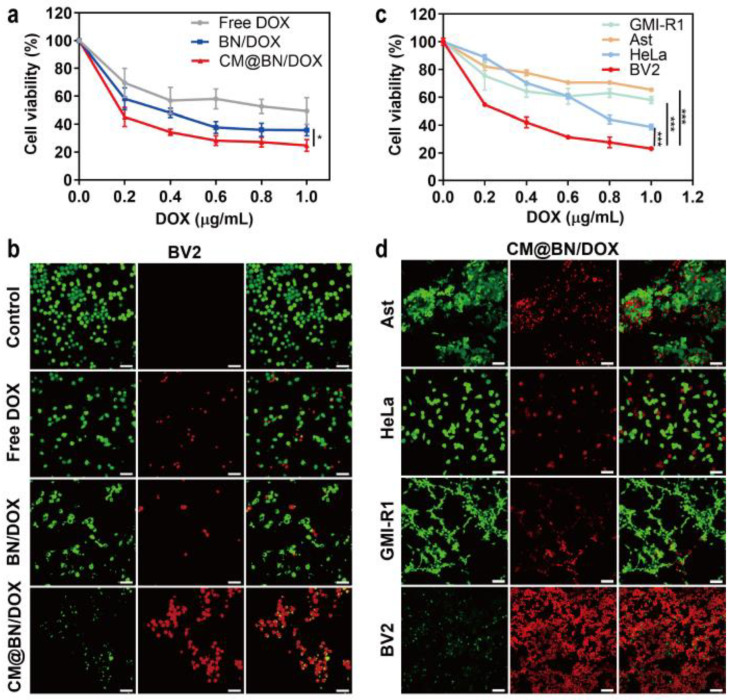
In vitro antitumor activity. (**a**) The viability of BV2 cells following treatment with varying concentrations of free DOX, BN/DOX, and CM@BN/DOX by the CCK-8 method. (**b**) The cytotoxicity of BV2 cells exposed to various treatments (green: living cells; red: dead cells). Scale bar = 100 µm. (**c**) The impact of varying concentrations of CM@BN/DOX on the cell viability of four different types of cells: BV2, HeLa, GMI-R1, and Ast. (**d**) The cytotoxicity of BV2, HeLa, GMI-R1, and Ast cells after treatment with CM@BN/DOX. Scale bar = 100 µm. Data are expressed as means ± SD, * *p* < 0.05, *** *p* < 0.001.

After observing promising results regarding the uniform uptake of cells, we proceeded to evaluate the effectiveness of the antitumor properties in vitro. Free DOX, BN/DOX, and CM@BN/DOX were cocultured with BV2 cells for 24 h. All three systems were operated with the same concentration of DOX. As shown in Figure 7a, the decrease of cell viability in all treatment groups was concentration-dependent, while under the same drug concentration, CM@BN/DOX-treated cancer cells showed significantly lower cell viability. It was expected that mono-dispersity and homologous cell identification of CM@BN/DOX enhanced the antitumor effect in vitro. These results were further confirmed by the live–dead cell staining shown in Figure 7b. Moreover, the therapeutic efficacy of CM@BN/DOX on BV2, HeLa, GMI-R1, and Ast cells was investigated. The results of Figure 7c,d showed that CM@BN/DOX was more effective in inhibiting homotypic BV2 cells compared to other types of cells. In vitro, the enhanced cytotoxicity of CM@BN/DOX to homogeneous cancer cells was mainly due to their excellent self-recognition internalization by the source cancer cell lines.

**Figure 8:**
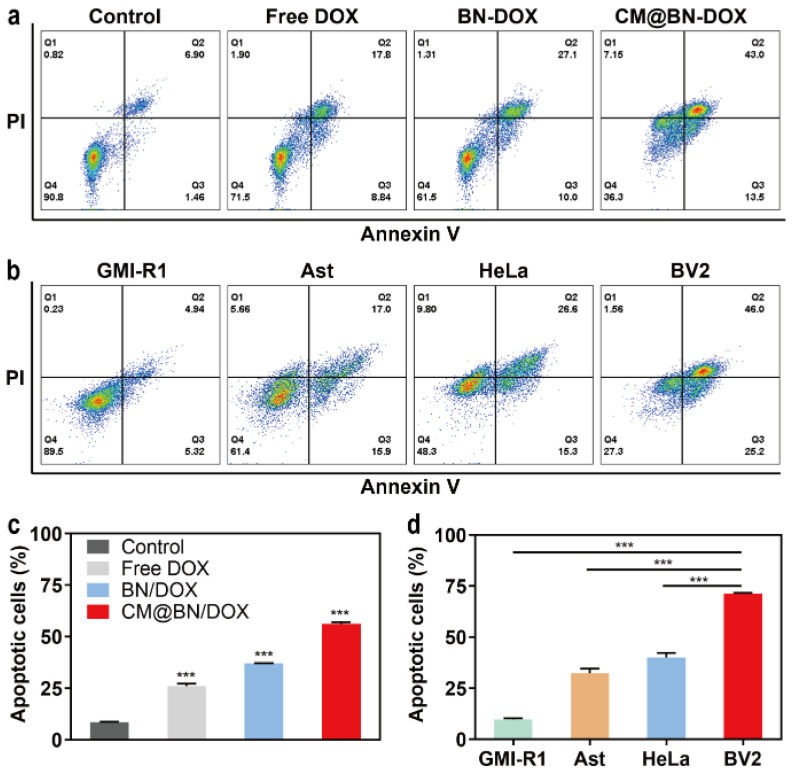
In vitro antitumor activity. (**a**,**c**) Cellular apoptosis of BV2 cells dealt with various reagents by FCM. (**b**,**d**) Cellular apoptosis of BV2, HeLa, GMI-R1, and Ast cells treated with CM@BN/DOX by FCM. Data are expressed as means ± SD, *** *p* < 0.001.

Further validation of the therapeutic effect of CM@BN/DOX on cancer cells was conducted using FCM. As illustrated in Figure 8a and c, BV2 cells treated with free DOX, BN/DOX, and CM@BN/DOX experienced a different apoptosis rate of 26.6%, 37.1%, and 56.5%, respectively. For different cells treated with CM@BN/DOX, the homologous cell BV2 had the highest apoptosis rate (71.2%) among groups (GMI-R1 cells: 10.3%, Ast cells: 32.9%, HeLa cells: 41.9%) (Figure 8b,d). The decreased cell growth and increased cancer cell apoptosis demonstrated the outstanding ability for tumor inhibition of CM@BN/DOX in vitro. The cell membrane endued BN nanoparticles with homologous targeting, which further enhanced their endocytosis by cancer cells, and the intracellular micro-acid environment promoted the drug release and enhanced the anti-tumor efficacy of CM@BN/DOX.

## 4. Conclusions

In this study, a cancer cell membrane-encapsulated BN nanoparticle with a core-shell structure was developed for homogeneous cancer therapy. The cell membrane coating improved the biocompatibility and enabled the excellent dispersity of BN. CM@BN/DOX exhibited favorable mono-dispersity, drug-loading capacity, and controlled drug release profile. Furthermore, membrane camouflage promoted the cellular internalization of CM@BN/DOX by homotypic cancer cells, which further enhanced homogeneous tumor eradication with negligible side effects. Therefore, with its excellent dispersibility, high loading capacity, and homogeneous targeting ability, CM@BN/DOX may provide a potential nanoplatform to improve intracellular drug transport and target tumor therapy. Given the promising therapeutic effect of CM@BN/DOX in vitro and their potential use in boron neutron capture therapy, further in vivo experiments are worthy for exploring their antitumor effect.

## Data Availability

The article included all the data and contributions presented in the study. For any additional inquiries, please direct them to the corresponding author.

## References

[1] Chugh V., Vijaya Krishna K., Pandit A. (2021). Cell Membrane-Coated Mimics: A Methodological Approach for Fabrication, Characterization for Therapeutic Applications, and Challenges for Clinical Translation. ACS Nano.

[2] Dang Y., Guan J. (2020). Nanoparticle-based drug delivery systems for cancer therapy. Smart Mater. Med..

[3] Senapati S., Mahanta A.K., Kumar S., Maiti P. (2018). Controlled drug delivery vehicles for cancer treatment and their performance. Signal Transduct. Target. Ther..

[4] Bhullar S., Goyal N., Gupta S. (2022). Synthesizing and Optimizing Rutile TiO_2_ Nanoparticles for Magnetically Guided Drug Delivery. Int. J. Nanomed..

[5] Zhang X., He C., Liu X., Chen Y., Zhao P., Chen C., Yan R., Li M., Fan T., Altine B. (2020). One-pot synthesis of a microporous organosilica-coated cisplatin nanoplatform for HIF-1-targeted combination cancer therapy. Theranostics.

[6] Su Y., Wang K., Li Y., Song W., Xin Y., Zhao W., Tian J., Ren L., Lu L. (2018). Sorafenib-loaded polymeric micelles as passive targeting therapeutic agents for hepatocellular carcinoma therapy. Nanomedicine.

[7] Zamboni W.C. (2008). Concept and clinical evaluation of carrier-mediated anticancer agents. Oncologist.

[8] Tong Q., Qiu N., Ji J., Ye L., Zhai G. (2020). Research Progress in Bioinspired Drug Delivery Systems. Expert Opin. Drug Deliv..

[9] Zhu L., Zhong Y., Wu S., Yan M., Cao Y., Mou N., Wang G., Sun D., Wu W. (2022). Cell membrane camouflaged biomimetic nanoparticles: Focusing on tumor theranostics. Mater. Today Bio.

[10] Zhao Y., Xie R., Yodsanit N., Ye M., Wang Y., Wang B., Guo L.W., Kent K.C., Gong S. (2021). Hydrogen peroxide-responsive platelet membrane-coated nanoparticles for thrombus therapy. Biomater. Sci..

[11] Yong T., Zhang X., Bie N., Zhang H., Zhang X., Li F., Hakeem A., Hu J., Gan L., Santos H.A. (2019). Tumor exosome-based nanoparticles are efficient drug carriers for chemotherapy. Nat. Commun..

[12] Li Q., Huang Z., Wang Q., Gao J., Chen J., Tan H., Li S., Wang Z., Weng X., Yang H. (2022). Targeted immunomodulation therapy for cardiac repair by platelet membrane engineering extracellular vesicles via hitching peripheral monocytes. Biomaterials.

[13] Ailuno G., Baldassari S., Lai F., Florio T., Caviglioli G. (2020). Exosomes and Extracellular Vesicles as Emerging Theranostic Platforms in Cancer Research. Cells.

[14] Lu M., Huang Y. (2020). Bioinspired exosome-like therapeutics and delivery nanoplatforms. Biomaterials.

[15] Liu H., Su Y.Y., Jiang X.C., Gao J.Q. (2023). Cell membrane-coated nanoparticles: A novel multifunctional biomimetic drug delivery system. Drug Deliv. Transl. Res..

[16] Zhao X., Yan C. (2022). Research Progress of Cell Membrane Biomimetic Nanoparticles for Tumor Therapy. Nanoscale Res. Lett..

[17] Hussain Z., Rahim M.A., Jan N., Shah H., Rawas-Qalaji M., Khan S., Sohail M., Thu H.E., Ramli N.A., Sarfraz R.M. (2021). Cell membrane cloaked nanomedicines for bio-imaging and immunotherapy of cancer: Improved pharmacokinetics, cell internalization and anticancer efficacy. J. Control. Release.

[18] Malhotra S., Dumoga S., Singh N. (2022). Red blood cells membrane-derived nanoparticles: Applications and key challenges in their clinical translation. Wiley Interdiscip. Rev. Nanomed. Nanobiotechnol..

[19] Malhotra S., Dumoga S., Joshi A., Mohanty S., Singh N. (2021). Polymeric micelles coated with hybrid nanovesicles enhance the therapeutic potential of the reversible topoisomerase inhibitor camptothecin in a mouse model. Acta Biomater..

[20] Fan L., Wei A., Gao Z., Mu X. (2023). Current progress of mesenchymal stem cell membrane-camouflaged nanoparticles for targeted therapy. Biomed. Pharmacother..

[21] Khosravi N., Pishavar E., Baradaran B., Oroojalian F., Mokhtarzadeh A. (2022). Stem cell membrane, stem cell-derived exosomes and hybrid stem cell camouflaged nanoparticles: A promising biomimetic nanoplatforms for cancer theranostics. J. Control. Release.

[22] Zhang T., Liu H., Li L., Guo Z., Song J., Yang X., Wan G., Li R., Wang Y. (2021). Leukocyte/platelet hybrid membrane-camouflaged dendritic large pore mesoporous silica nanoparticles co-loaded with photo/chemotherapeutic agents for triple negative breast cancer combination treatment. Bioact. Mater..

[23] Wang D., Gao C., Zhou C., Lin Z., He Q. (2020). Leukocyte Membrane-Coated Liquid Metal Nanoswimmers for Actively Targeted Delivery and Synergistic Chemophotothermal Therapy. Research.

[24] Zhang W., Gong C., Chen Z., Li M., Li Y., Gao J. (2021). Tumor microenvironment-activated cancer cell membrane-liposome hybrid nanoparticle-mediated synergistic metabolic therapy and chemotherapy for non-small cell lung cancer. J. Nanobiotechnol..

[25] Fan Y., Cui Y., Hao W., Chen M., Liu Q., Wang Y., Yang M., Li Z., Gong W., Song S. (2021). Carrier-free highly drug-loaded biomimetic nanosuspensions encapsulated by cancer cell membrane based on homology and active targeting for the treatment of glioma. Bioact. Mater..

[26] Wang Y., Zhao Z., Liu C., Hao M., Kong C., Zhao X., Gao Y., Zhang Y., Cui W., Zhang C. (2022). B16 Membrane-Coated Vesicles for Combined Photodynamic Therapy and Immunotherapy Shift Immune Microenvironment of Melanoma. Int. J. Nanomed..

[27] Fan R., He S., Wang Y., Qiao J., Liu H., Galstyan L., Ghazaryan A., Cai H., Feng S., Ni P. (2022). Targeted delivery of a PROTAC induced PDEδ degrader by a biomimetic drug delivery system for enhanced cytotoxicity against pancreatic cancer cells. Am. J. Cancer Res..

[28] Liu Y., Sukumar U.K., Kanada M., Krishnan A., Massoud T.F., Paulmurugan R. (2021). Camouflaged Hybrid Cancer Cell-Platelet Fusion Membrane Nanovesicles Deliver Therapeutic MicroRNAs to Presensitize Triple-Negative Breast Cancer to Doxorubicin. Adv. Funct. Mater..

[29] Ihsanullah I. (2021). Boron nitride-based materials for water purification: Progress and outlook. Chemosphere.

[30] Pan D., Su F., Liu H., Ma Y., Das R., Hu Q., Liu C., Guo Z. (2020). The Properties and Preparation Methods of Different Boron Nitride Nanostructures and Applications of Related Nanocomposites. Chem. Rec..

[31] Türkez H., Arslan M.E., Sönmez E., Açikyildiz M., Tatar A., Geyikoğlu F. (2019). Synthesis, characterization and cytotoxicity of boron nitride nanoparticles: Emphasis on toxicogenomics. Cytotechnology.

[32] Sharker S.M. (2019). Hexagonal Boron Nitrides (White Graphene): A Promising Method for Cancer Drug Delivery. Int. J. Nanomed..

[33] Li X., Zhi C., Hanagata N., Yamaguchi M., Bando Y., Golberg D. (2013). Boron nitride nanotubes functionalized with mesoporous silica for intracellular delivery of chemotherapy drugs. Chem. Commun..

[34] Ailuno G., Balboni A., Caviglioli G., Lai F., Barbieri F., Dellacasagrande I., Florio T., Baldassari S. (2022). Boron Vehiculating Nanosystems for Neutron Capture Therapy in Cancer Treatment. Cells.

[35] Barth R.F., Mi P., Yang W. (2018). Boron delivery agents for neutron capture therapy of cancer. Cancer Commun..

[36] Wang W., Lin J., Xing C., Chai R., Abbas S., Song T., Tang C., Huang Y. (2017). Fe_3_O_4_ nanoparticle-coated boron nitride nanospheres: Synthesis, magnetic property and biocompatibility study. Ceram. Int..

[37] Nakamura H., Koganei H., Miyoshi T., Sakurai Y., Ono K., Suzuki M. (2015). Antitumor effect of boron nitride nanotubes in combination with thermal neutron irradiation on BNCT. Bioorg. Med. Chem. Lett..

[38] Niskanen J., Zhang I., Xue Y., Golberg D., Maysinger D., Winnik F.M. (2016). Boron nitride nanotubes as vehicles for intracellular delivery of fluorescent drugs and probes. Nanomedicine.

[39] Zhang H., Feng S., Yan T., Zhi C., Gao X.D., Hanagata N. (2015). Polyethyleneimine-functionalized boron nitride nanospheres as efficient carriers for enhancing the immunostimulatory effect of CpG oligodeoxynucleotides. Int. J. Nanomed..

[40] Weng Q., Wang B., Wang X., Hanagata N., Li X., Liu D., Wang X., Jiang X., Bando Y., Golberg D. (2014). Highly water-soluble, porous, and biocompatible boron nitrides for anticancer drug delivery. ACS Nano..

[41] Feng S., Zhang H., Yan T., Huang D., Zhi C., Nakanishi H., Gao X.D. (2016). Folate-conjugated boron nitride nanospheres for targeted delivery of anticancer drugs. Int. J. Nanomed..

[42] Feng S., Li H., Ren Y., Zhi C., Huang Y., Chen F., Zhang H. (2020). RBC membrane camouflaged boron nitride nanospheres for enhanced biocompatible performance. Colloids Surf. B Biointerfaces.

[43] Tang C., Bando Y., Huang Y., Zhi C., Golberg D. (2008). Synthetic Routes and Formation Mechanisms of Spherical Boron Nitride Nanoparticles. Adv. Funct. Mater..

[44] Shen J.W., Li C., Yang M.Y., Lin J.F., Yin M.D., Zou J.J., Wu P.Y., Chen L., Song L.X., Shao J.W. (2022). Biomimetic nanoparticles: U937 cell membranes based core-shell nanosystems for targeted atherosclerosis therapy. Int. J. Pharm..

[45] Li J.Q., Zhao R.X., Yang F.M., Qi X.T., Ye P.K., Xie M. (2022). An erythrocyte membrane-camouflaged biomimetic nanoplatform for enhanced chemo-photothermal therapy of breast cancer. J. Mater. Chem. B.

[46] Yang J., Zhang L., Zhou Q., Chen F., Stenzel M., Gao F., Liu C., Yuan H., Li H., Jiang Y. (2021). Self-assembled anionic and cationic Au nanoparticles with Au nanoclusters for the exploration of different biological responsiveness in cancer therapy. Nanoscale Adv..

[47] Verma A., Stellacci F. (2010). Effect of surface properties on nanoparticle-cell interactions. Small.

[48] Pradipta A.R., Ahmadi P., Terashima K., Muguruma K., Fujii M., Ichino T., Maeda S., Tanaka K. (2021). Targeted 1,3-dipolar cycloaddition with acrolein for cancer prodrug activation. Chem. Sci..

[49] Omar M.M., Hasan O.A., Zaki R.M., Eleraky N.E. (2021). Externally Triggered Novel Rapid-Release Sonosensitive Folate-Modified Liposomes for Gemcitabine: Development and Characteristics. Int. J. Nanomed..

[50] Matthews A.T., Soni H., Robinson-Freeman K.E., John T.A., Buddington R.K., Adebiyi A. (2021). Doxorubicin-Induced Fetal Mesangial Cell Death Occurs Independently of TRPC6 Channel Upregulation but Involves Mitochondrial Generation of Reactive Oxygen Species. Int. J. Mol. Sci..

[51] Sui J., He M., Yang Y., Ma M., Guo Z., Zhao M., Liang J., Sun Y., Fan Y., Zhang X. (2020). Reversing P-Glycoprotein-Associated Multidrug Resistance of Breast Cancer by Targeted Acid-Cleavable Polysaccharide Nanoparticles with Lapatinib Sensitization. ACS Appl. Mater. Interfaces.

